# Characterization and Functional Analysis of the Potato Pollen-Specific Microtubule-Associated Protein SBgLR in Tobacco

**DOI:** 10.1371/journal.pone.0060543

**Published:** 2013-03-25

**Authors:** Chen Liu, Xin Qi, Qian Zhao, Jingjuan Yu

**Affiliations:** State Key Laboratory for Agro-biotechnology, College of Biological Sciences, China Agricultural University, Beijing, China; University of Heidelberg Medical School, Germany

## Abstract

Microtubule-associated proteins play a crucial role in the regulation of microtubule dynamics, and are very important for plant cell and organ development. SBgLR is a potato pollen-specific protein, with five imperfect V-V-E-K-K-N/E-E repetitive motifs that are responsible for microtubule binding activity. In present study, SBgLR showed typical microtubule-associated protein characteristics; it bound tubulin and microtubules, and colocalized with microtubules *in vitro*. We also found that SBgLR could form oligomers, and that both the SBgLR monomers and oligomers bundle microtubules *in vitro*. Constitutive expression of SBgLR in tobacco caused curving and right-handed twisting root growth, abnormal directional cell expansion and cell layer arrangement, and pollen abortion. Immunofluorescence staining assays revealed that microtubule organization is altered in root epidermal cells in SBgLR-overexpressing lines. These suggest that SBgLR functions as a microtubule-associated protein in pollen development. Our results indicate that normal organization of MTs may be crucial for pollen development.

## Introduction

Microtubules (MTs) play a crucial role in plant cell growth and development. They participate in many cellular processes including cell division, expansion, cell shape determination, material intracellular transport, signal transduction and chromosome partitioning [1]–[5]. The regulation of MT dynamics facilitates the reorganization of MTs during different cell phases, and the response to the environmental perturbations in different organs [6]–[8]. Many microtubule-associated proteins (MAPs) have been reported to function in the regulation of MTs in higher plants. The balance of MAP activity is crucial for the normal organization of MTs. Many abnormal MAP activities may disturb the MT dynamic, and result in dramatic effects on MT organization, cell morphogenesis, and even organ chirality [9]. For example, the mutant of the *Arabidopsis thaliana* gene *SKU6/SPIRAL1*, which encodes a plus end-localized MT-interacting protein, shows right-skewed root growth, right-handed twisting of roots, and abnormal hypocotyls and leaf petioles [10]. The *mor1/gem1* mutant shows increased cortical MT stability, and defects in MT organization lead to organ twisting and isotropic cell expansion in roots [11]–[12]. In *Arabidopsis*, the gene responsible for the *fra2* mutation encodes a katanin-like protein, and the *fra2* mutant shows aberrant cortical MT orientation and reduced cell elongation [13].

Several MAP motifs responsible for MT-binding have been identified. The K-K-E-E and K-K-E-I/V repetitive motifs are reported to be involved in MT-binding activity in mouse [14]. In plants, V-V-E-K-K-N/E-E repetitive motifs are conserved, and are responsible for MT-binding activity in *Arabidopsis* and *Solanaceae*. The *Arabidopsis MAP18* gene encodes seven V-E-E-K-K repetitive motifs, and functions in directional cell growth by destabilizing cortical MTs [15]. A class of *Solanaceae* pollen-specific proteins, containing imperfect V-V-E-K-K-N/E-E repetitive motifs, have been reported to interact with MTs *in vitro* and *in vivo*. The SB401 protein isolated from wild potato (*Solanum berthaultii*) has six V-V-E-K-K-N/E-E repetitive motifs; it binds and bundles MTs and F-Actin *in vitro* and/or *in vivo* [16]. The *St901* gene, isolated from *Solanum tuberosum*, encodes a protein of 217 amino acid residues. This protein contains five V-V-E-K-K-N/E-E imperfect repetitive motifs, and shares 79.51% identity with SB401. The suppression of the *St901* gene in potato results in aberrant pollen at maturation [17]. Another protein TSB, cloned from *Solanaceae lycopersicum* also contains V-V-E-K-K-N/E-E motifs [18]. However, with the exception of SB401, the activities of these proteins on MTs, and their functions in pollen development are still unknown. In wild potato, the *SBgLR* gene encodes a hydrophilic protein of 211 amino acid residues, rich in Lys and Glu [19], and encodes five imperfect V-V-E-K-K-N/E-E repetitive motifs.

Here, we further investigate the activities of SBgLR on MT regulation, and its role in pollen development. We found that the recombinant SBgLR binds to both tubulin and MTs *in vitro*. Furthermore, the SBgLR protein could form oligomers on a Native-PAGE gel. Both the SBgLR monomers and the oligomers bundle MTs, suggesting that there are more than two binding sites within the SBgLR sequence. Specific phenotypes and cell shapes in different organs were investigated through overexpressing SBgLR in tobacco. The SBgLR-overexpressing tobacco plants showed the same typical morphology and cell shapes in roots, hypocotyls and cotyledon pavement cells as seen in MAP defective mutants. Pollen abortion was observed in transgenic tobacco plants, suggesting that SBgLR interacts with MTs and regulates their organization. Therefore, SBgLR plays an important role in potato pollen development.

## Materials and Methods

### Ethics Statement

The Committee of Experiment Animals of China Agricultural University approved the protocols we used in our study for antibody production and tubulin purification. The rabbit was raised in standardized pathogen-free conditions in the Animal Care Facility at Beijing B&M Biotech Co., Ltd. Blood was drawn from the marginal ear vein under anesthesia to ameliorate suffering. For tubulin purification, porcin brain was taken from the slaughtered pigs, which were used as food material, from the No. 5 slaughtering company in Beijing.

### Sequence comparison

The amino acid sequences of St901 (*S. tuberosum* cv. Desiree AY526087), TSB (*S. lycopersicum* AAM53961), MAP18 (*Arabidopsis thaliana* BAC41928), SB401 (*S. berthaultii* CAA65228), and SBgLR (*S. tuberosum* AAR29265) were blasted using the DNAMAN 6.0 software package (LynnonBiosoft). The imperfect repetitive V-V-E-K-K-N/E-E motifs were shaded in gray.

### Purification of recombinant SBgLR protein and preparation of its antibody

The full-length coding sequence of SBgLR was cloned into pET30a vector (Novagen), and transformed into *Escherichia coli* strain BL21 (DE3). Bacteria were cultured in LB liquid medium containing 500 mg/L kanamycin, to OD_600_ = 0.5, and then 50 mM isopropyl-β-d-thiogalactoside (IPTG) was added to induce the expression of the recombinant protein for 4 h. The bacteria were then centrifuged at 7 000 *g* at 4°C for 10 min, and resuspended using lysate buffer (50 mM NaH_2_PO_4_, 300 mM NaCl, 20 mM imidazole, pH 8.0). After sonicating of the turbid liquid, the lysis solution was centrifuged at 30 000 *g* at 4°C for 30 min. The suspension was added to the Ni-NTA agarose resin column (GE Healthcare, Sweden) and washed with lysate buffer containing 50, 100 and 150 mM imidazole. The recombinant protein was eluted with 250 mM imidazole elution buffer, and dialyzed against PEM buffer (0.1 M PIPES, 1 mM EGTA, 1 mM MgSO_4_, pH 6.9) at 4°C overnight. The purified protein was stored at −80°C for MT assay, or injected into a rabbit to elicit antiserum. The antiserum was purified using the Protein A resin column, and the cyanogen bromide resin column (Amersham, Pharmacia Biotech).

### Purification and polymerization of tubulin

The tubulin was purified from porcine brain according to a previously described method [20]. The tubulin was polymerized into MTs before use. After centrifuging the tubulin at 70 000 *g* at 4°C for 30 min, 10 µl GTP (10 mM) and 90 µl tubulin was incubated at 37°C for 20 min, and then 2 µM, 20 µM and 200 µM taxol was added to the tubulin, and polymerized for 15 min at each concentration of taxol. The polymerized MTs were centrifuged at 13 523 *g* at 25°C for 20 min and washed with PEMT (0.1 M PIPES, 1 mM EGTA, 1 mM MgSO_4_, 20 mM taxol, pH 6.9) twice.

### Tubulin and MT-binding assay

For the tubulin binding assay 2, 4, 8 and 16 µM of proteins (SBgLR, tubulin and BSA) in total volumes of 5 µl were spotted onto PVDF membranes (Millipore Corporation, USA) and air-dried. The membranes were then incubated with 3% BSA in TBST (50 mM Tris, 150 mM NaCl and 0.05% Tween20, pH 7.5) at 4°C overnight. The membranes were then incubated with 10 µM SBgLR, or 10 µM tubulin at room temperature for 2 h, and washed three times for 10 min; they were then incubated with anti-SBgLR or anti-β-tubulin antibody at the dilution of 1∶1 000 and 1∶500, respectively, at room temperature for a further 2 h. Following another washing with TBST, the membranes were incubated with the alkaline phosphatase-conjugated goat anti-rabbit or anti-mouse IgG (1∶5000). The bounded proteins were detected using NBT/BCIP color development reagent (Promega, USA).

For MT co-sedimentation assays, recombinant SBgLR and BSA were centrifuged at 75 000 *g* for 30 min at 4°C before use. 0 to 12 µM recombinant SBgLR protein, 12 µM BSA, and 4 µM taxol-stabled MTs (tsMTs) was added to a 50 µl reaction volumes in PEMT buffer. Following a 30 min reaction at room temperature, samples were centrifuged at 75 000 *g* for 30 min at 25°C. The pellet was rinsed with PEMT, and resuspended using sample loading buffer (10 mM Tris-HCl pH 8.0, 10% glycerol, 5% β-mercaptoethanol, 2% SDS, 0.1% bromophenol). The samples were analyzed by 8% SDS-PAGE, and the gel was stained with Coomassie Brilliant Blue R250. The amounts of the recombinant SBgLR bound to MTs were determined by gel scanning, and the binding ratio between the recombinant SBgLR and MTs was analyzed by the Image J 2.1.4.6 analysis system (National Institutes of Health).

### MT-bundling assay and electron microscopy

For the MT-bundling assay, 0.5 µM taxol-stabled rhodamine-labeled MTs (tsrMTs) and 2 µM recombinant SBgLR were incubated at 25°C for 30 min, and then the mixture was fixed with 1% glutaraldehyde. The same amounts of tsrMTs without or with boiling denatured recombinant SBgLR were set as negative controls. Subsequently, a 1 µl sample was placed on the slide, and examined using a confocal microscope (SP5, Leica, Germany). Photographs were taken using a Leica AF Hardware configurator (Leica).

For the negative-staining assay, 0.5 µM tsMTs and 2 µM recombinant SBgLR were incubated at 25°C for 30 min; the same amounts of boiling denatured recombinant SBgLR were used as a negative control. Then the mixture was adsorbed on formvar-coated copper grids for 10 min, stained with 2% uranyl acetate for 3 min, and air-dried. The samples were examined with a Hitachi 7500 electron microscope (Japan). Photographs were taken using iTEM (OSIS, Germany).

For transmission electron microscopy (TEM), 0.5 µM tsMTs and 2 µM recombinant SBgLR were incubated at 25°C for 30 min, then centrifuged at 75 000 *g* at 25°C for 30 min. The pellet was fixed with fixation buffer (50 mM PBS, 1% glutaraldehyde, 4% paraformaldehyde and 5 mM EGTA, pH 6.8) at 4°C for 1 h, and post-fixed with 1% osmium tetroxide at 4°C for 30 min. The sample was dehydrated using an ethanol gradient, embedded in Spurr's resin, and sectioned into 90 nm thick slices. The sections were stained with 2% uranyl acetate and 2.66% lead citrate, and observed using a transmission electron microscope (7500, Hitachi) operated at 80 kV. Photographs were taken using iTEM (OSIS, Germany).

### Co-localization of SBgLR and MTs

The 0.5 µM tsrMTs and 2 µM recombinant SBgLR were incubated at 25°C for 30 min. Next, the sample was incubated with antibody against SBgLR (1∶500) at room temperature for another 1 h. After incubation, the sample was labeled with FITC-conjugated goat anti-rabbit IgG secondary antibody (1∶100, Sigma-Aldrich, USA) at room temperature for 30 min. After being fixed with 1% glutaraldehyde, the sample was examined under a confocal microscope (SP5, Leica) at wavelengths of 488 nm and 561 nm, to visualize SBgLR and MTs, respectively. Incubation of boiling denatured recombinant SBgLR with tsrMTs was carried out as a negative control. Images were taken using the Leica AF hardware configurator (Leica).

### Native-PAGE analysis

Purified natural and 0.5% β-mercaptoethanol treated recombinant SBgLR protein was separated by 12% native polyacrylamide gel. The gel was stained with Coomassie brilliant blue R-250 (Amresco, USA). The molecular weight of the oligomers were estimated according to the equation log_10_ M_W_ = −bx+k (M_W_, molecular weight; x, relative migration rate; b and k, constant).

### Plant transformation and molecular analysis

The vector p35S::SBgLR was constructed by cloning the full-length coding sequence of *SBgLR* into pCambia2300. This was transformed into *Agrobacterium tumefaciens* strain LBA4404. Tobacco transformation was carried out using the leaf disc method [21]. The transgenic plants were confirmed by PCR amplification using *SBgLR* (ORF) specific primer pair: Primer 1 5′-GAAAGTGGTGGCTGTGGAAA-3′, and Primer 2 5′-AATTGGCTTGAT GGTCTCCT-3′. For RT-PCR amplification, total RNA from T_2_ seedlings was extracted using TRIzol reagent (CWBIO, China), and the cDNA first strand synthesis was carried out according to the protocol of the Reverse Transcription System (A3500, Promega). RT-PCR amplification was performed using the same primer pair (Primer 1; Primer 2) used for PCR amplification.

For western blot analysis, 5 µg of proteins from different tissues were separated on 12% SDS-PAGE, and transferred to PVDF membrane (Millipore Corporation). The membrane was incubated with anti-SBgLR (1∶5 000) or anti-actin primary antibody (1∶500), and the alkaline phosphatase-conjugated goat anti-rabbit or anti-mouse IgG secondary antibody (1∶5000). The signals were detected with NBT/BCIP reagent (Promega).

### Light microscopy (LM) and scanning electron microscopy (SEM)

For LM, cotyledons and hypocotyls were fixed in FAA (5% acetic acid, 5% formaldehyde solution, and 63% ethanol) at 4°C for 48 h. After dehydrating in an ethanol series of 50%, 70%, 80%, 90%, 95% and 100%, the samples were vitrified in a dimethylbenzene/ethanol series of 33%, 50%, 66% and 100%, and then embedded in paraffin. The embedded blocks were sectioned with a microtome (Leica) to a thickness of 10 µm. The sections were stained with 0.5% safranin and 0.5% fast green, and observed with a light microscope (BX51, Olympus, Japan).

For SEM, cotyledons, hypocotyls and pollen grains were observed using a scanning electron microscope (TM3000, Hitachi). For the measurements of hypocotyl epidermal cells, data was collected from at least 60 cells, and collected from two different hypocotyls. For the observation of cotyledon pavement cells, observations were consistently conducted on cells located in the mid-region of the cotyledon, at the same developmental stage. Data were obtained from the measurement of at least 170 cells, collected from four different cotyledons, and from three individual seedlings.

### Immunofluorescence staining of MTs

Immunofluorescence staining assay was performed for the observation of MTs in roots. Roots were cut from 7-day-old seedlings and fixed in PEM containing 4% (w/v) paraformaldehyde at room temperature for 1 h. A mouse anti-β-tubulin monoclonal antibody (Sigma-Aldrich) at 1∶800 dilution was applied as primary antibody, and TRITC-conjugated goat anti-mouse IgG antibody at 1∶500 dilution (Sigma-Aldrich) was used as secondary antibody. Confocal microscopy observations and image acquisition were performed using a Leica SP5 confocal microscope.

### Pollen I_2_-KI staining

Pollen grains were harvested from fresh opened anthers on a microscopic slide, and stained with I_2_-KI solution (0.5% I_2_ and 3% KI). They were observed with a light microscope (BX51, Olympus). For each line, pollen grains from at least four anthers and from two flowers were observed; pollens were observed under six fields of visions for statistical analysis.

## Results

### Blast analysis of SBgLR and purification of the recombinant SBgLR protein

**Figure 1:**
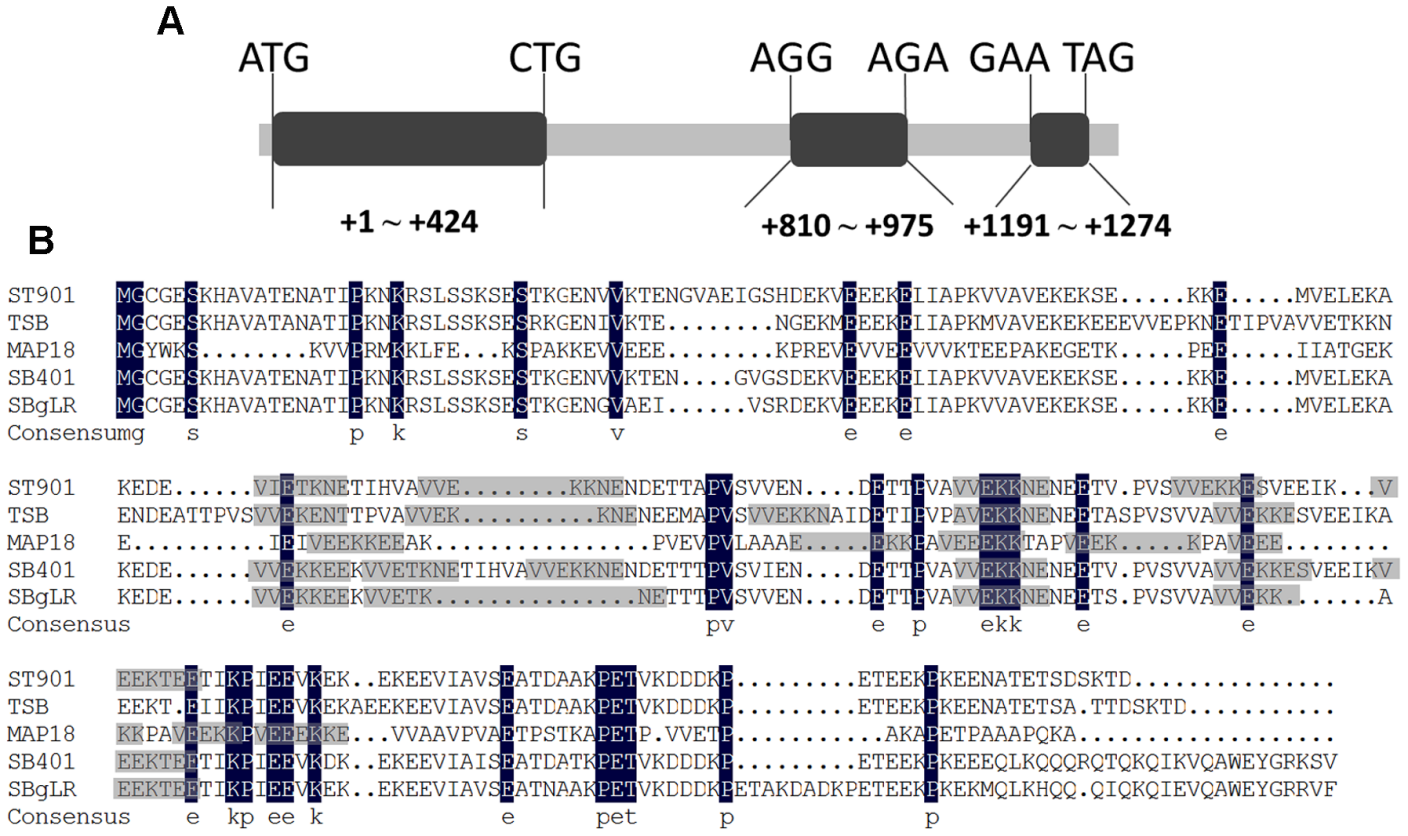
Gene structure of *SBgLR* and amino acid comparison of SBgLR and its homologs. A, Gene structure of *SBgLR*. Three exons were illustrated in black. B, Amino acid comparison between SBgLR and its homologs: St901, TSB, MAP18 and SB401. The conserved motifs that responsible for MT-binding domain were shadowed in gray.

**Figure 2:**
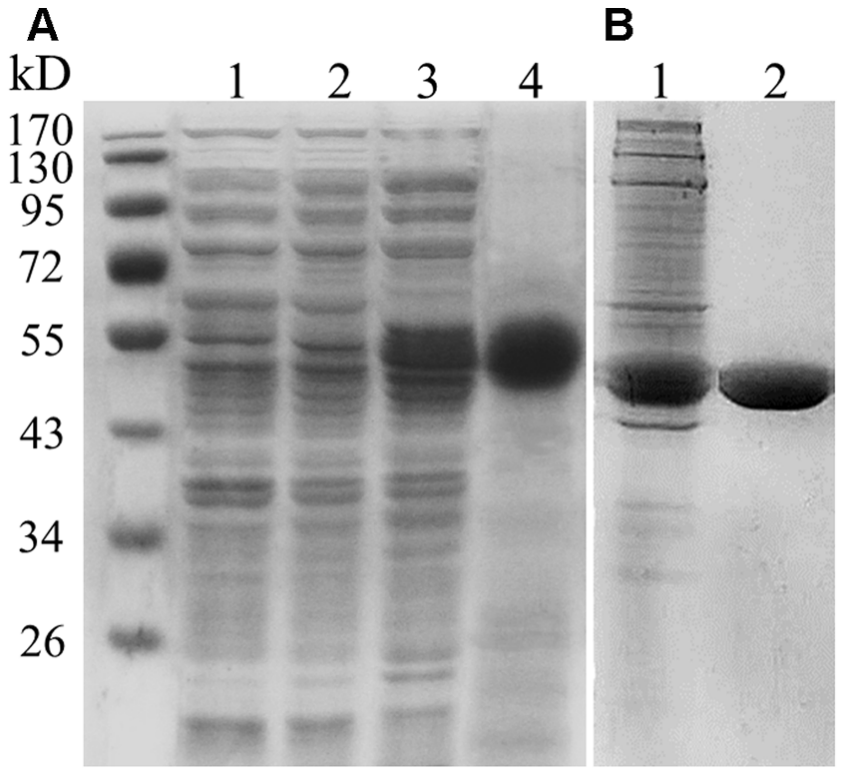
Coomassie brilliant blue stained gels of recombinant SBgLR protein and tubulin. A, Lane 1, 15 µg of total extract from bacteria cells containing pET30a empty vector without IPTG induction; Lane 2, 15 µg of total extract from bacterial cells containing pET30a-SBgLR vector without induction; Lane 3, 20 µg of total extract from bacteria cells containing pET30a-SBgLR vector with 50 mM IPTG induction for 4 h; Lane 4, 10 µg purified SBgLR recombinant protein. B, Lane 1, crude extract of porcine tubulin; Lane 2, purified porcine tubulin. The sizes of protein marker were labeled in the ‘kD’ column.

*SBgLR* is a pollen-specific gene containing three exons and two introns (Fig. 1A), its corresponding protein has an estimated molecular mass of 23.23 kDa, and a pI of 4.33 [19], [22]. The full-length cDNA of *SBgLR* encodes a protein with five V-V-E-K-K-N/E-E repetitive motifs; these are conserved in *Solanum* and *Arabidopsis* (Fig. 1B), and are responsible for MT-binding activity. To investigate the binding activity of SBgLR on MTs, we cloned the full-length coding sequence of *SBgLR* into the pET30a vector, to produce recombinant SBgLR protein. The 6×His-SBgLR recombinant protein was analyzed by SDS-PAGE. The molecular weight of the recombinant protein was approximately 50 kDa (Fig. 2A), and it showed a reduction of mobility compared with the predicted molecular weight of 30 kDa. Other proteins with V-V-E-K-K-N/E-E motifs (including MAP1B, SB401 and MAP18) show a similar reduction in mobility [14], [15], [23].

### SBgLR binds tubulin and MTs *in vitro*

**Figure 3:**
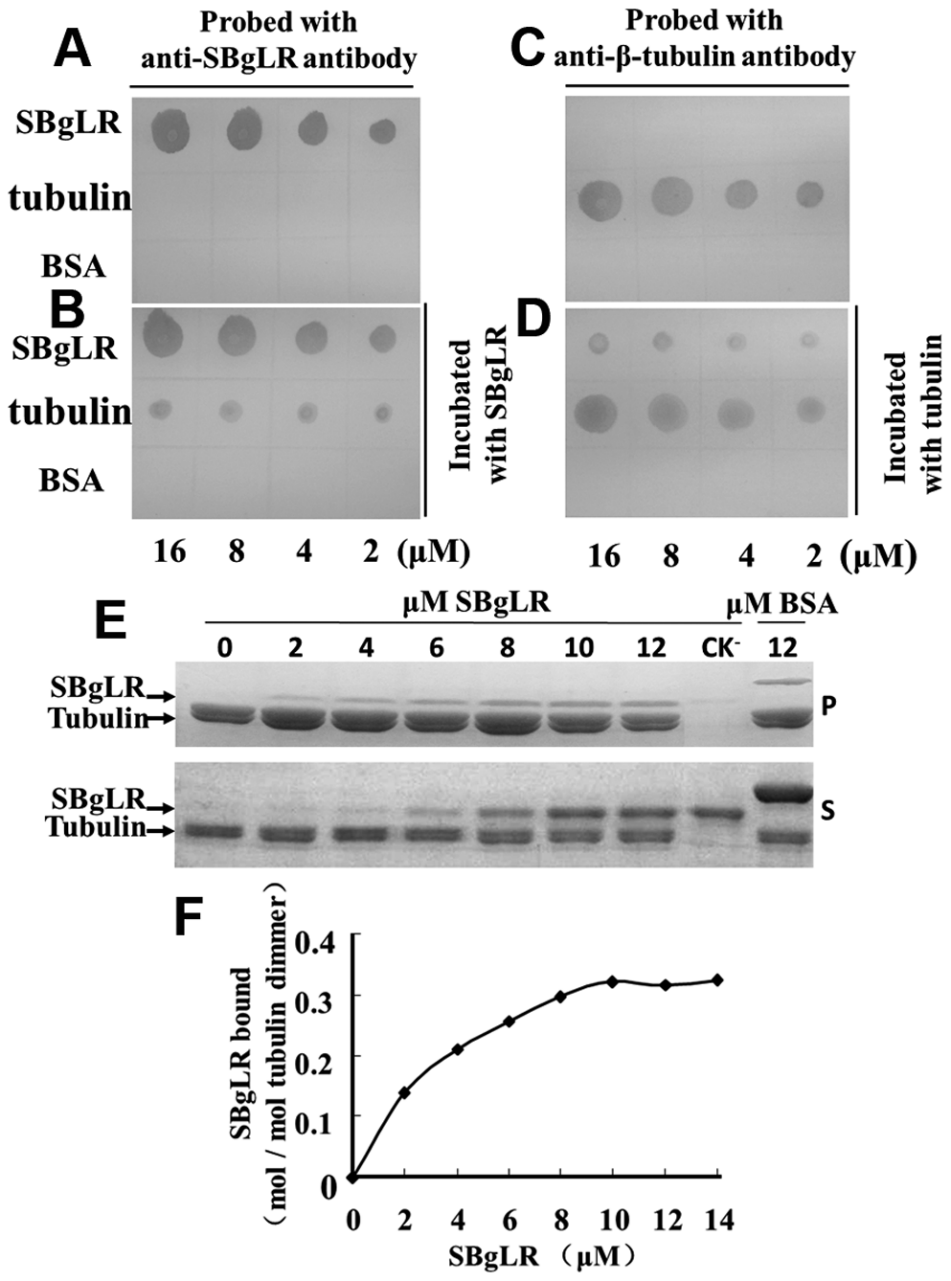
The recombinant SBgLR binds tubulin and MTs *in vitro*. A to D, Immunoblotting assay. Aliquots of 2, 4, 8, and 16 µM of SBgLR, tubulin, and BSA were spotted onto PVDF membranes. The membrane was preincubated with (B) or without (A) 10 µM recombinant SBgLR, and then probed with anti-SBgLR antibody. The membrane was preincubated with (D) or without (C) 10 µM tubulin, and then probed with anti-β-tubulin antibody. E and F, The co-sedimentation assay. E, Coomassie brilliant blue stained gels of the pellets (P) and the supernatant (S) from the co-sedimentation assay. The recombinant SBgLR protein mainly appeared in the supernatant (S) after the centrifugation in the absence of tsMTs and co-precipitated with tsMTs into the pellets (P) when tsMTs was added. BSA (12 µM) was used as a negative control. F, Quantitative analysis of the binding between recombinant SBgLR and tsMTs. The binding of the recombinant SBgLR protein to MTs was saturated at a ratio of ∼0.35 mole recombinant SBgLR per mole tubulin dimer estimated by gel scanning.

To determine whether the recombinant SBgLR binds to tubulin directly, the immunoblotting assays were performed using the recombinant SBgLR (Fig. 2A), and the purified porcine tubulin (Fig. 2B). In these assays, 2, 4, 8 and 16 µM of proteins (recombinant SBgLR, tubulin and BSA) were spotted onto four PVDF membranes, and incubated with the recombinant SBgLR or tubulin solutions. The incubated membranes were then hybridized with anti-SBgLR or anti-β-tubulin antibodies. The anti-SBgLR antibody recognized both the SBgLR (upper line in Fig. 3A and 3B) and the tubulin spots following incubation with 10 µM recombinant SBgLR (middle line in Fig. 3B), whereas only the SBgLR, and not the tubulin spots, were detected when incubation with recombinant SBgLR was omitted (middle line in Fig. 3A). Similarly, the anti-β-tubulin antibody recognized the tubulin (middle line in Fig. 3C and 3D) and the SBgLR spots following incubation with 10 µM tubulin (upper line in Fig. 3D), but no SBgLR spots were detected in the absence of tubulin incubation (upper line in Fig. 3C). Various concentrations of BSA were used as negative controls, and neither of the antibodies recognized the BSA spots (bottom line in Fig. 3A to 3D). We observed that when the concentration of recombinant SBgLR and tubulin was increased, that there was an increased affinity of the recombinant SBgLR to tubulin (Fig. 3B and 3D). These results suggested that the recombinant SBgLR binds to tubulin *in vitro* directly and specifically.

Co-sedimentation assays were performed to determine whether the recombinant SBgLR protein could bind to MTs *in vitro*. Various concentrations of recombinant SBgLR protein co-precipitated with tsMTs in the presence of tsMTs, whereas no recombinant SBgLR protein was detected from the pellet of MT-free control (Fig. 3E). To further determine the binding affinity of the recombinant SBgLR to tsMTs, different quantities of recombinant SBgLR proteins (0 to 12 µM) were incubated with tsMTs. As the amount of recombinant SBgLR increased in the reaction systems, an increase in sedimentation following ultra-centrifugation was observed (Fig. 3E). The binding ratio was 0.35 (mole/mole, SBgLR/tubulin dimmers) at saturation, based on four independent experiments (Fig. 3F). This confirmed that the recombinant SBgLR binds to MTs *in vitro*.

### SBgLR monomers and oligomers bundle MTs *in vitro*

**Figure 4:**
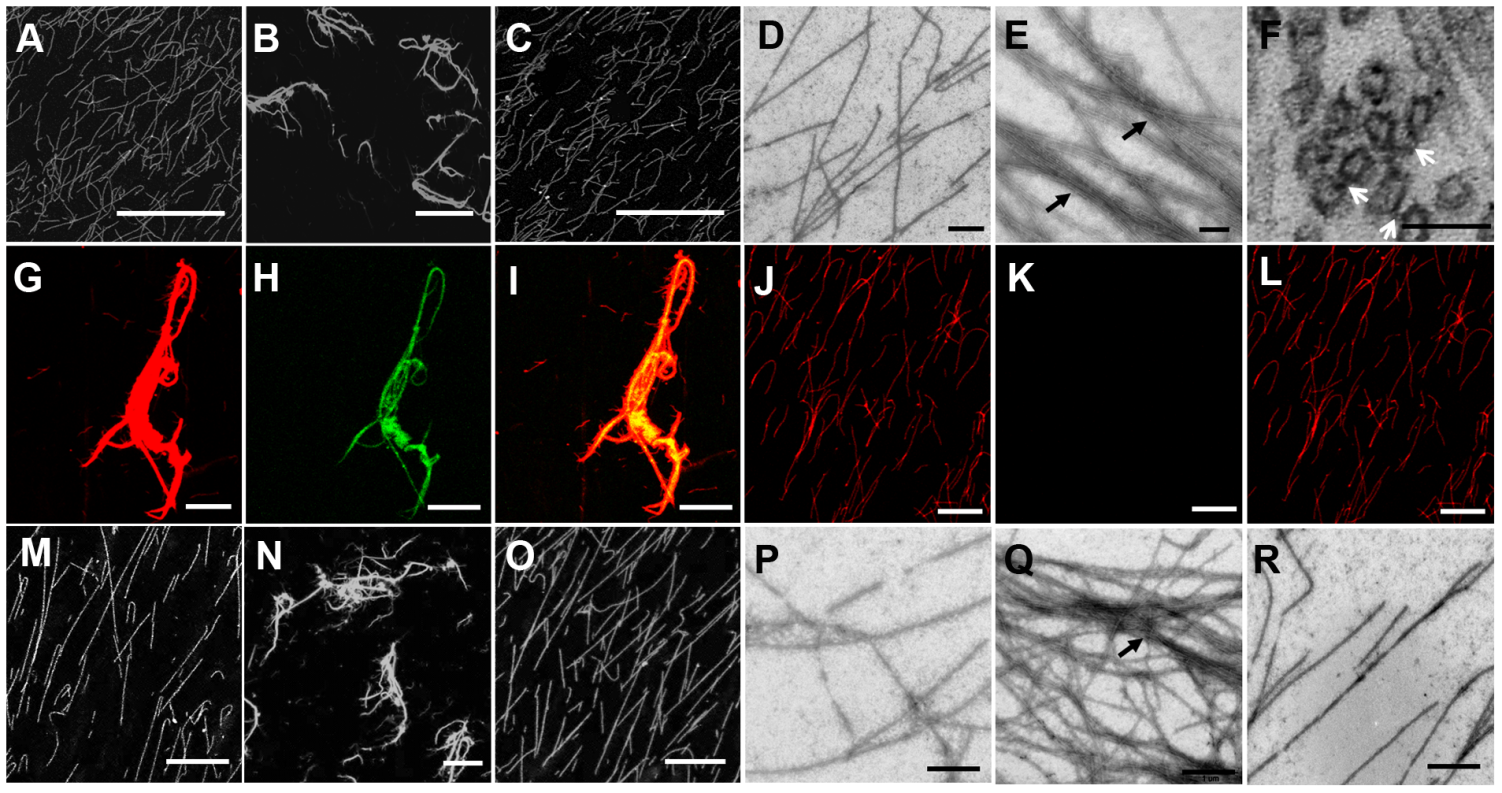
SBgLR protein bundles MTs *in vitro*. A to C, Confocal microscopy of recombinant SBgLR on MT-bundling. A, tsrMTs. Single-filament tsrMTs were scattered throughout the solution, no MT-bundles were observed. B, MT-bundles were formed when recombinant SBgLR was added. C, boiling denatured recombinant SBgLR was added as a negative control. Bar = 25 µm from A to C. D and E, Negative-staining electron microscopy of recombinant SBgLR on MT-bundling. D, tsMTs. Bar = 1 µm. E, Scattered tsMTs formed aggregates and integrated into a meshwork (black arrows) when native recombinant SBgLR was added. Bar = 100 nm. F, Longitudinal section of the MT-bundles. The cross-bridges were observed between the tsMTs (white arrows). Bar = 200 nm. G to L, Co-localization assay using confocal microscopy. G to I, tsrMTs were incubated with recombinant SBgLR, and then incubated with anti-SBgLR primary antibody and FITC conjugated goat anti-rabbit IgG secondary antibody. G, tsrMTs; H, recombinant SBgLR; I, Merged image of G and H. J to L, tsrMTs were incubated with boiling denatured recombinant SBgLR as a negative control. No MT-bundles and SBgLR signals were detected. J, tsrMTs; K, SBgLR; L, merged image of J and K. Bar = 10 µm from G to L. M to O, Confocal microscopy of recombinant SBgLR monomers on MT-bundling. M, tsrMTs. N, MT-bundles were formed when β-mercaptoethanol-treated recombinant SBgLR was added to tsrMTs. O, boiling denatured β-mercaptoethanol-treated recombinant SBgLR was added to tsrMTs as a negative control. Bar = 25 µm from M to O. P to R, Negative-staining electron microscopy of β-mercaptoethanol-treated recombinant SBgLR on MTs-bundling. P, tsMTs. Q, Scattered tsMTs formed aggregates (black arrows) when β-mercaptoethanol-treated recombinant SBgLR was added. R, boiling denatured β-mercaptoethanol-treated recombinant SBgLR was added to tsMTs as a negative control. Bar = 1 µm from P to R.

We subsequently examined the MT-bundling activity of the purified recombinant SBgLR protein using confocal microscopy and electron microscopy. The results revealed that the organization of tsrMTs remain unchanged when recombinant SBgLR was absent or denatured (Fig. 4A and 4C). However, tsrMTs became disconnected bundles in the presence of recombinant SBgLR (Fig. 4B). We further confirmed this specific activity of recombinant SBgLR for bundling tsMTs using negative-staining electron microscopy. In the presence of recombinant SBgLR, the scattered tsMTs formed aggregates and integrated into a meshwork (Fig. 4E). The tsMTs within the aggregates were organized into a parallel arrangement (black arrow in Fig. 4E). This indicated that the recombinant SBgLR bundles MTs *in vitro*. To further examine the structure of SBgLR-induced MT-bundles, we observed the cross-bridges within MT-bundles using TEM. Cross-bridges between tsMTs were observed from the transection (white arrows in Fig. 4F). Subsequently, the co-localization results revealed that the recombinant SBgLR protein induced tsrMTs into MT-bundles when 2 µM SBgLR was incubated with 0.5 µM tsrMTs (Fig. 4G to 4I). Furthermore, the recombinant SBgLR protein colocalized with MT-bundles (Fig. 4I). In contrast, MT-bundles and SBgLR-MTs co-localization signal were not observed when denatured recombinant SBgLR was added to the reaction system (Fig. 4J to 4L). This indicated that the recombinant SBgLR binds MTs, and organized them into MT-bundles *in vitro*.

**Figure 5:**
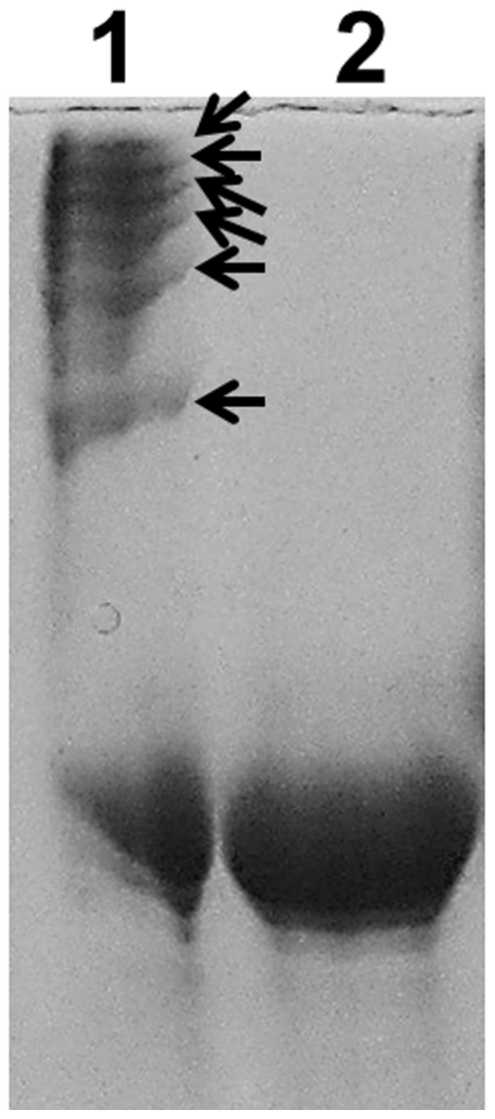
Native-PAGE analysis of recombinant SBgLR protein. Lane 1, Native recombinant SBgLR protein without β-mercaptoethanol treated. Arrows indicated SBgLR oligomers; Lane 2, Purified recombinant SBgLR protein treated with 0.5% β-mercaptoethanol.

When bundling MTs, MAPs can form oligomers, use more than two MT-binding sites, or both. To investigate the way in which recombinant SBgLR bundled MTs, we examined the polymerizing activity of recombinant SBgLR using Native-PAGE. Seven bands were seen on the native gel with the estimated molecular weights of 25.23 kDa, 51.00 kDa, 60.25 kDa, 65.90 kDa, 72.01 kDa, 76.36 kDa and 79.35 kDa (Fig. 5, lane 1 and Fig. S2). However, when the recombinant SBgLR was treated with 0.5% β-mercaptoethanol, only the band with the lowest molecular weight was detected (Fig. 5, lane 2).

To further investigate the bundling activity when recombinant SBgLR is reduced to monomers, MT-bundling and negative-staining assays were performed. The use of confocal microscopy revealed that single tsrMTs were induced into MT-bundles when 0.5% β-mercaptoethanol-treated recombinant SBgLR was added to the tsrMTs (Fig. 4N). Furthermore, negative-staining electron microscopy results supported the data obtained from the MT-bundling assay. Figure 4Q shows that in the presence of β-mercaptoethanol-treated recombinant SBgLR, the scattered tsMTs formed MT-bundles. No MT-bundles were observed in the absence of β-mercaptoethanol-treated recombinant SBgLR or in the presence of boiling denatured β-mercaptoethanol-treated recombinant SBgLR (Fig. 4P and 4R). This suggested the presence of more than two MT-binding sites within the SBgLR sequence.

As a consequence, both SBgLR oligomers and monomers could bundle MTs *in votro*.

### SBgLR-overexpressing tobacco lines show abnormal phenotypes and cell growth

**Figure 6:**
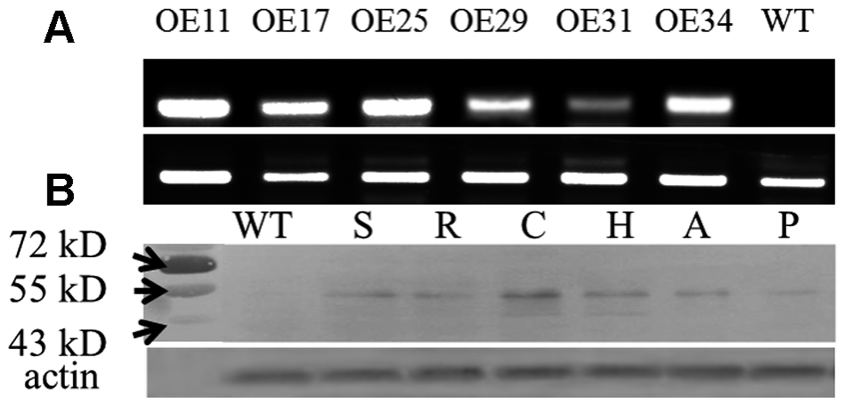
Analysis of *SBgLR* expression in different transgenic lines. A, Semi-quantitative RT-PCR analysis. The cDNAs reverse transcribed using RNA extracted from 7-day-old seedlings of different transgenic lines were used. The tobacco actin gene was used as a reference gene. B, Immunoblotting analysis of SBgLR accumulation in the stem (S), root (R), cotyledon (C), hypocotyl (H), anther (A) and pollen (P) of transgenic tobacco. The actin protein was detected as a loading control.

To analyze the function of SBgLR *in vivo*, the full-length coding sequence of the *SBgLR* gene was transformed into tobacco. In total, thirty-five transgenic lines were obtained. The 7-day-old seedlings from six transgenic lines were taken randomly for quantification of *SBgLR* transcript by RT-PCR analysis. The specific products were amplified from all six transgenic lines (Fig. 6A). The transgenic lines 11 (OE11) and 25 (OE25), which had higher *SBgLR* transcription levels were selected for further study (Fig. 6A). Meanwhile, immunoblotting was performed on OE25 for qualification and quantification of SBgLR protein in different tissues. SBgLR was detected at similar levels in the stems, roots and anthers; higher in cotyledons and hypocotyls; and lower in pollen grains. No SBgLR protein was detected in the WT control (Fig. 6B).

**Figure 7:**
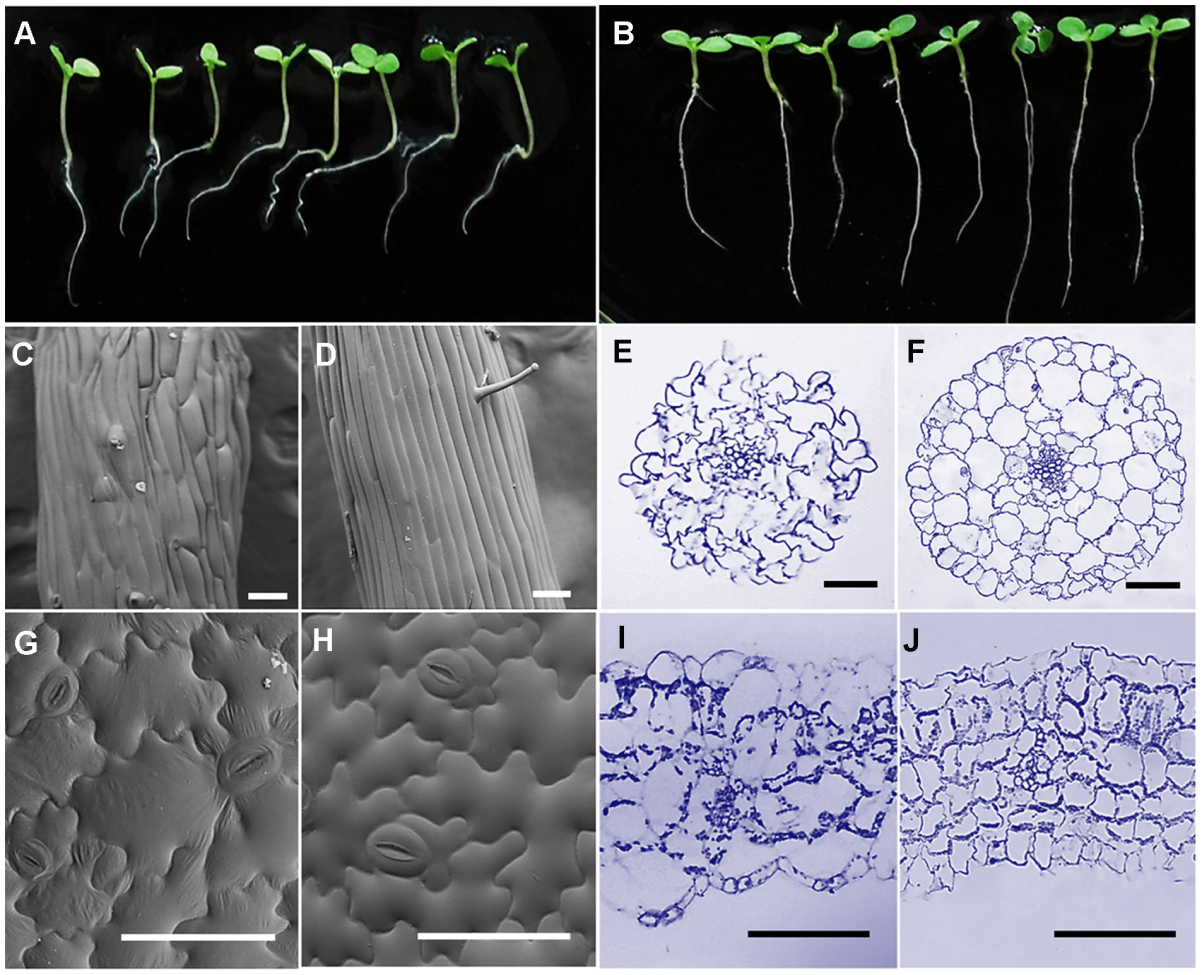
Phenotype and cell morphology observation of SBgLR-overexpressing tobacco. A and B, Phenotype of the seedlings. A, OE25; B, WT. C and D, SEM images of hypocotyl epidermal cells. C, OE25; D, WT. Bar = 100 µm. E and F, Hypocotyl cross sections. E, OE25; F, WT. Bar = 100 µm. G and H, SEM images of cotyledon pavement cells. G, OE25; H, WT. Bar = 100 µm. I and J, Cotyledon sections. I, OE25; J, WT. Bar = 100 µm.

Microtubules play key roles in cell morphogenesis [5], and a given MAP activity may cause changes in cell shape or organ chirality [9]. In this study, we ectopically expressed *SBgLR* in tobacco to explore if there were specific phenotypes and morphologies in SBgLR-overexpressing lines. The examination of these lines revealed that the overexpression of *SBgLR* gave rise to abnormal root growth, and directional cell expansion (Fig. 7). When grown on the surface of solid medium containing 1% agar, the roots of SBgLR-overexpressing lines were curved, and showed a right-handed twisting pattern, and were shorter than those of WT plants (Table 1; Fig. 7A). No curving or twisting patterns were observed in WT plants (Fig. 7B). The hypocotyls of transgenic lines were longer than those seen in WT plants (Table. 1).

**Table 1:** Statistical analysis of hypocotyl and cotyledon pavement cells.

Line	Hypocotyl			Cotyledon Pavement Cell			
	LR (mm)	LH (mm)	LHEC ( µm)	NCPC/mm^2^	CL ( µm)	NW ( µm)	LL ( µm)
WT	25±2	6.1±0.6	170±20	240±34	130±16	27±3	19±4
	(*n* = 32)	(*n* = 32)	(*n* = 60)	(*n* = 284)	(*n* = 270)	(*n* = 196)	(*n* = 237)
OE	13±1^**^	9.1±1.6^*^	140±10	170±12^**^	120±10	49±5^**^	18±3
	(*n* = 40)	(*n* = 40)	(*n* = 96)	(*n* = 273)	(*n* = 208)	(*n* = 172)	(*n* = 225)

Data are means ± SD (number of measurements). LR, Length of Roots; LH, Length of Hypocotyls; LHEC, Length of Hypocotyl Epidermal Cells; NCPC, Number of Cytoledon Pavement Cell; CL, Cell Length; NW, Neck Width; LL, Lobe Length. The significance of difference was evaluated by Student's *t*-test, (^*^ *p*<0.05; ^**^ *p*<0.01).

Abnormal cell shape and growth were also observed in hypocotyls and cotyledon pavement cells when using LM and SEM. SEM revealed that the epidermal cells of SBgLR-overexpressing hypocotyls were swelling, irregularly arranged and formed a bumpy surface (Fig. 7C). Hypocotyl epidermal cells were shorter than those of WT plants (Table 1). LM revealed that the hypocotyl cell layers of SBgLR-overexpressing lines were disordered (Fig. 7E). In addition to the hypocotyl epidermal cells, the cortex cells also displayed a jagged pattern (Fig. 7E). However, no abnormalities were observed in WT hypocotyl cells (Fig. 7D and 7F).

The cotyledon epidermal pavement cells of WT tobacco displayed a jigsaw-puzzle-like shape (Fig. 7H), with lobes (Fig. S1) intercalated into adjacent cells (Fig. 7H). However, the cotyledon epidermal pavement cells of SBgLR-overexpressing lines were nearly rectangular or round in shape (Fig. 7G). The mid-region of the cotyledon pavement cells of SBgLR-overexpressing tobacco plants were larger in neck width than that seen in WT pavement cells (Table 1), but no dramatic changes in cell length (CL) and lobe length (LL) were observed (Table 1). Statistical analysis revealed that there were 239.6±34.4 pavement cells per mm^2^ in WT cotyledons, whereas, only 167.2±12.2 pavement cells per mm^2^ were observed in SBgLR-overexpressing cotyledons (Table 1). LM of cotyledon sections showed that both the epidermal and the cortex cells were intumescent and irregularly arranged in SBgLR-overexpressing lines (Fig. 7I) in comparison with WT plants (Fig. 7J).

**Figure 8:**
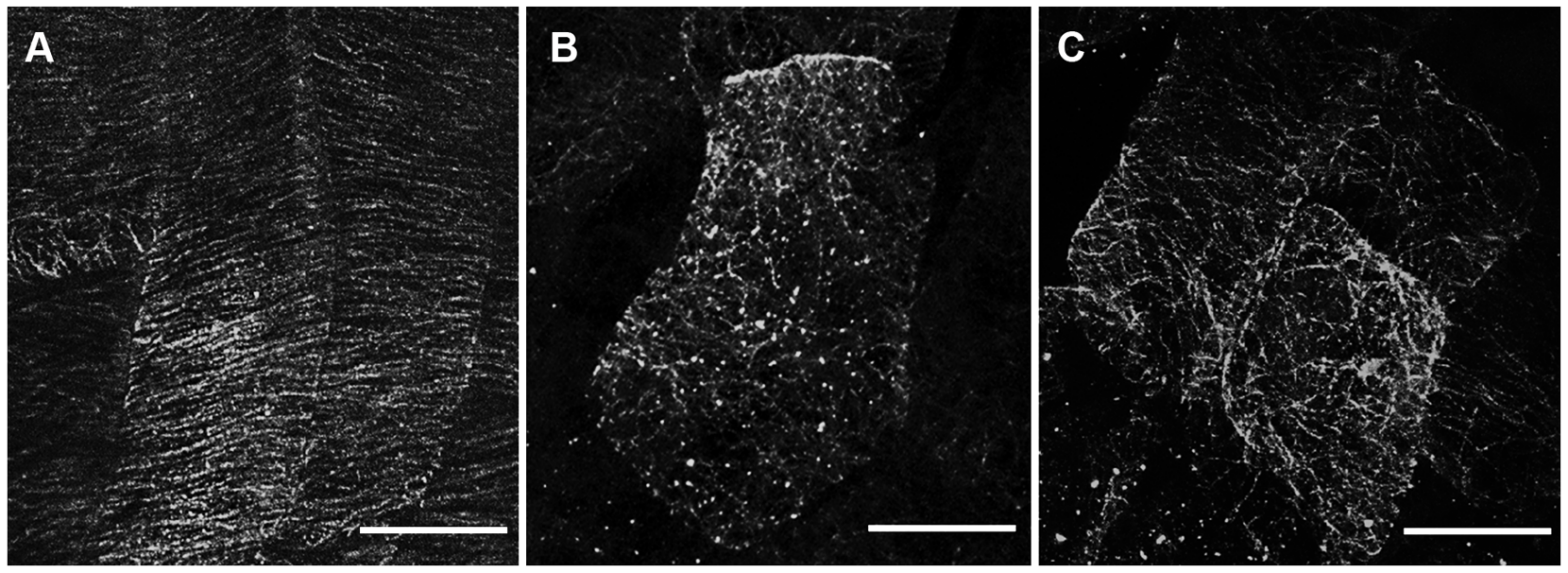
Immunofluorescence staining of cortical MTs in roots. The cortical MTs of root epidermal cells visualized by immunofluorescence microscopy. A, WT. The cortical MTs were transverse arranged. B. OE11; C, OE25. The cortical MTs in OE11 and OE25 were mostly organized into oblique or longitudinal MT arrays. Bar = 10 µm from A to C.

To investigate the correlation between MT organization and the abnormal phenotypes of SBgLR-overexpressing lines, we used immunofluorescence staining for observation of MTs. Because the chloroplast autofluorescence made it difficult to observe MTs in cotyledon pavement cells, we used immunofluorescence microscopy to visualize cortical MTs in root epidermal cells. The root epidermal cells of 7-day-old WT seedlings had transverse cortical MTs (Fig. 8A). However, the cortical MTs in root epidermal cells overexpressing SBgLR were mostly organized into oblique or longitudinal MT arrays (Fig. 8B and 8C). Our observations indicated that the overexpression of SBgLR resulted in the abnormal organization of cortical MTs, and consequently, abnormalities in cell morphogenesis occurred.

All these results suggested that SBgLR interferes with the normal organization of MTs in SBgLR-overexpressing lines, leading to abnormal cell growth in different organs. Therefore, we infer that SBgLR may be crucial for MT organization during pollen development.

### Ectopic expression of SBgLR causes pollen abortion in transgenic tobacco

**Figure 9:**
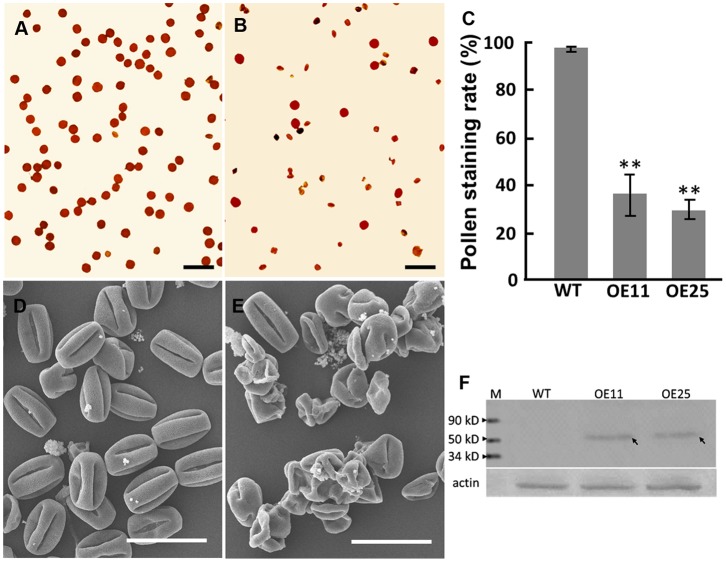
Pollen grain analysis and the SBgLR accumulation in pollen grain. A and B, I_2_-KI staining of pollen grain. A, WT; B, OE25. Bar = 100 µm. C, Statistical data of I_2_-KI staining rate. Data are means ± SD, Student's *t* -test, ^**^ *p*<0.01; D and E, SEM of pollen grains. D, WT; E, OE25. Bar = 50 µm. F, Immunoblotting analysis of SBgLR in both transgenic and WT pollen grain. The weak signal was detected in OE11 and OE25 (indicated by arrows).

The fact that *SBgLR* is specifically expressed in potato pollen suggests an important role in pollen development. To investigate the function of SBgLR in pollen development, we used I_2_-KI staining assays to observe the phenotypes of transgenic and WT pollen. The pollen grains of WT plants were round and dark stained (Fig. 9A). However, most of the transgenic pollen grains were wizened and unstained (Fig. 9B), suggesting that there is a deficiency of storage materials in SBgLR-overexpressing pollens. Statistical analyses of pollen staining rate revealed that the percentage of normal pollen in the WT was 98.77%, but it was only 36.83% and 29.79% in OE11 and OE25, respectively (Fig. 9C). This suggests that a severe pollen abortion occurs in these two transgenic lines. Further observation of pollen grains by SEM showed that the WT pollen grains were full (Fig. 9D), whereas the pollen grains of SBgLR-overexpressing lines had an invaginated and shrunken shape (Fig. 9E). Further, immunoblotting was carried out using proteins extracted from WT and transgenic mature pollen to confirm the expression of SBgLR in the transgenic pollen. A specific band of approximately 50 kDa was detected in the OE11 and OE25 pollens, but not in the WT pollen (Fig. 9F). This result suggested that the expression of SBgLR in tobacco disturbs the normal development of pollen grains.

## Discussion

### V-V-E-K-K-N/E-E motifs are conserved and responsible for MT-binding and bundling activities

The V-V-E-K-K-N/E-E is reported to be one of the domains responsible for MT-binding and/or bundling activities. It is highly conserved in *S. berthaultii*, *S. tuberosum*, *S. lycopersicum, Arabidopsis* and mouse [14], [15], [16]. Nobel *et al*. indicated that these kinds of motifs probably interact ionically with the negatively charged carboxyterminal portion of α-and/or β-tubulin, resulting in the binding of MAPs to tubulin or MTs [14]. The individual motif within one protein binds tubulin dimers independently, and this bridging of tubulin dimer within MTs stabilized MTs. SBgLR, like SB401 [16], binds and bundles MTs *in vitro*. MT-bundles are usually observed in MT structures in plant cells [24]. The presence of cross-bridges between MTs can be revealed by electron microscopy [25], [26]. In our study, the cross-bridges have been observed using TEM. The MAP65 family from tobacco BY-2 cells is important in MT-bundling [27]. The TMBP200, isolated from telophase tobacco BY-2 cells, has been reported to be responsible for MT-bundling *in vitro* [28]. For the bundling of MTs, MAPs either consists more than two MT-binding sites or are able to form an oligomer, or both. The Native-PAGE result revealed that recombinant SBgLR could form oligomers (Fig. 5). Similar results were obtained by use of recombinant AtMAP65-1 [29]. However, with the exception of the 51.00 kDa band, the molecular weight of the oligomers seems not to be an integral multiple of SBgLR monomer. We infer that the major reason for this phenomenon was that the protein was running on a native gel. That is to say, not only the molecular weight but also the protein shape and charge, which could not be excluded in Native-PAGE, determined the apparent molecular weight. Meanwhile, the SBgLR monomers also bundle MTs *in vitro*. This suggested that there are more than two MT-binding sites within SBgLR protein. Nobel *et al*. indicated proteins that exhibit more than two binding sites may cross-bridge tubulin dimers, be involved in nucleating MT polymerization, and play a role in the stabilizing MTs [14].

### Multiple organs show abnormal phenotypes and cell growth in SBgLR constitutive-expression lines

The shape of the plant cell is defined by the cell wall, which itself is mainly determined by the cytoskeleton. MT organization depends on a variety of MAPs. Several MAP defective mutants show abnormal cell morphology, growth and expansion [10]–[13], from which we can speculate the function of a MAP candidate. This gives us a way of investigating the interaction and regulation activities of a MAP candidate in cells through the analysis of changes in phenotype and cell shape, even when ectopic expression of a tissue-specific gene has been conducted. In this study, we observed cell morphology and shape in different organs. The roots of SBgLR-overexpressing tobacco lines showed right-handed twisting growth (Fig. 7A); this is similar to that seen in *Arabidopsis spr2-2* and *wnd2-1* mutants [30], [31]. In most cases, organ twisting may be attributable to deficiency in MT dynamics or organization [32]. Furthermore, right-handed twisting seems to occur in mutants, such as *spr2-2* and *wnd2-1*, when MT disassembly is inhibited [30], [31]. Cell shape and cell layer arrangement were greatly changed in OE11 and OE25 (Fig. 7C to 7J).

The abnormal phenotypes were correlated with abnormal MT organization (Fig. 8B and 8C). The results of MT-bundling assays indicated that both monomers and oligomers of SBgLR have MAP activities *in vitro* (Fig. 4). However, which was the functional form in plants was still needed to be discussed. The expression of SBgLR altered the normal organization of MTs in cells (Fig. 8) suggested that the function of SBgLR was achieved through its MAP activity. Since both forms of SBgLR have MAP activities, we infer that both of these two forms might have biological functions during potato pollen development. The probable roles of SBgLR momomers, dimers and oligomers in MT organization was showed in Figure S3. The SBgLR monomers organize the single MT into parallel arranged MT-bundles (Fig. S3A), and the presence of SBgLR dimers and oligomers could make the MT-bundles cross-linked or reoriented (Fig. S3B to S3D). At least, this structure was observed from negative-staining assay (Fig. 4E). Additionally, it may also mediate the stabilization of the paralleled MT-bundles (Fig. S3C) or mesh the MT-bundles into network (Fig. S3D). In potato pollen, some factors or interacting proteins may interact with SBgLR, through which the balance between SBgLR momomers, dimers and oligomers achieved, and established the stably and normally organized MTs. The balance between SBgLR monomers and oligomers, together with other MAPs, might be necessary for SBgLR during potato pollen development. But it should be noted that the molecular mechanism for the regulation of this balance, and the exact functions of different forms of SBgLR in potato pollen development are needed to be further studied.

Although the growth of SBgLR-overexpressing lines were influenced, they could still reach the reproductive growth stage, suggesting that cell shape and arrangement abnormalities were not lethiferous for the transgenic lines.

### SBgLR may play a role in pollen development through influencing the organization of MTs

SBgLR is a potato pollen-specific expression gene that has no homolog in tobacco. However, pollen abortion was observed in transgenic tobaccos when overexpression of the *SBgLR* gene was driven by the *CaMV35S* promoter (Fig. 9). The activity of *CaMV35S* promoter in reproductive tissues is inconclusive. Van der Leede-Plegt *et al*. [33] reported that no promoter activity has been detected in the mature pollen of tobacco and tomato. However, Twell *et al*. [34] and Wilkinson *et al*. [35] reported that low levels of GUS activity, driven by the *CaMV35S* promoter, could be detected in tobacco pollen. In our study, we detected the expression of *SBgLR* under the control of *CaMV35S* promoter through the use of immunoblotting analyses (Fig. 6B and 9F). This was a similar result to those found by Twell and Wilkinson [34], [35].

In SBgLR-overexpressing lines, microspore release from tetrads microspore was normal (Fig. 9). However, because of the lack of inclusion, pollen abortion occurred. This implies that there is a deficiency in material transition or deposition in SBgLR-overexpressing tobacco plants. Normal organization of MTs is crucial for material transportation and deposition in storage tissues. In the maize endosperm, zein deposition and protein body formation only occur when MTs and microfilaments are sufficient [36], [37]. RNA-related proteins that are involved in protein deposition may play a role in the regulation of MT organization. These include EF1α, a well-known regulator of translation in eukaryotes, which plays an important role in zein deposition and protein body formation in maize endosperm [38]–[42]. Similarly, SBgLR may interfere with the normal organization of MTs in transgenic pollen grains, resulting in the deficiency of pollen inclusion transportation or deposition in SBgLR- overexpressing tobacco.

Taken together, we infer that SBgLR regulates the organization of MTs in pollen, and the normal organization of MTs is critical for material deposition during pollen maturation.

## Supporting Information

Figure S1 **Schematic diagram of tobacco cotyledonal epidermis pavement cell.**
(TIF)

Figure S2 **Estimation of molecular weight of the recombinant SBgLR monomer and oligomer.** A, Native-PAGE analysis of the recombinant SBgLR. The native standard proteins were indicated by arrows. B, Molecular weight estimation for the oligomers. The molecular weights of the oligomers were 25.23 kDa, 51.00 kDa, 60.25 kDa, 65.90 kDa, 72.01 kDa, 76.36 kDa and 79.35 kDa, respectively. MW, Molecular weight; Rf, Protein migration. The molecular weight of the oligomer was estimated by the equation: log_10_MW = –1.2512x+4.9301 (x, protein migration).
(TIF)

Figure S3 **Schematic diagram of the probable roles of SBgLR monomers, dimers and oligomers in MT organization.** A, The SBgLR monomers organized single MT into MT-bundles. B, The SBgLR dimers and oligomers make the MT-bundles cross-linked or reoriented. C and D, The SBgLR proteins stabilize the paralleled MT-bundles or make the MT-bundles meshing into network.
(TIF)
