# Supplementary figures and images for: Characterization and Functional Analysis of the Potato Pollen-Specific Microtubule-Associated Protein SBgLR in Tobacco
Source: PLoS One. 2013 Mar 25;8(3):e60543. doi: 10.1371/journal.pone.0060543 (PMC3607588; doi:10.1371/journal.pone.0060543)

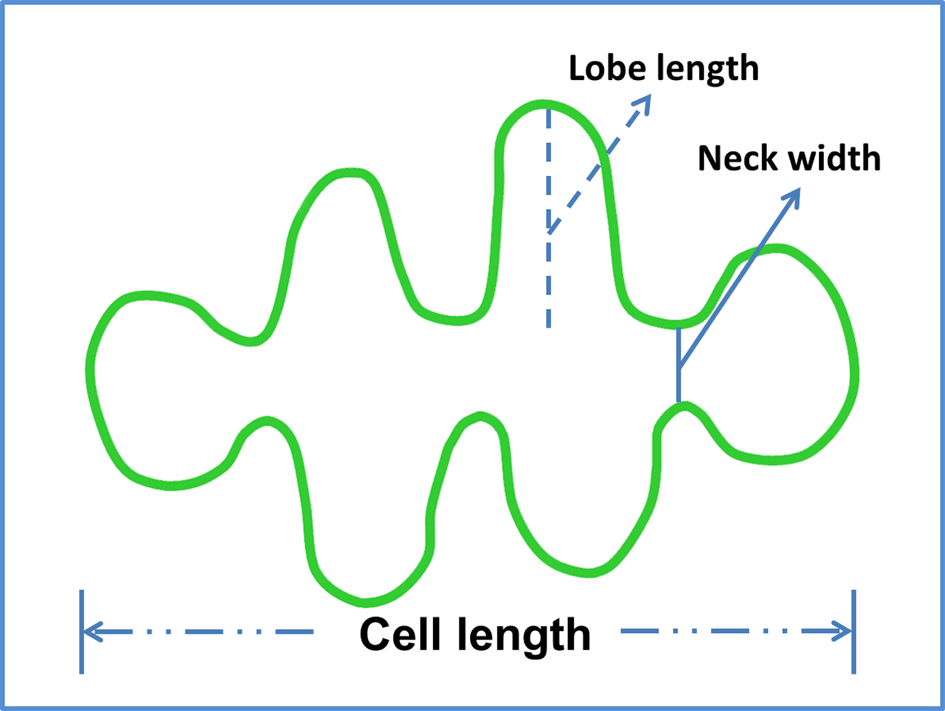

Supplement: Figure S1 — Schematic diagram of tobacco cotyledonal epidermis pavement cell. (TIF) [file pone.0060543.s001.tif]

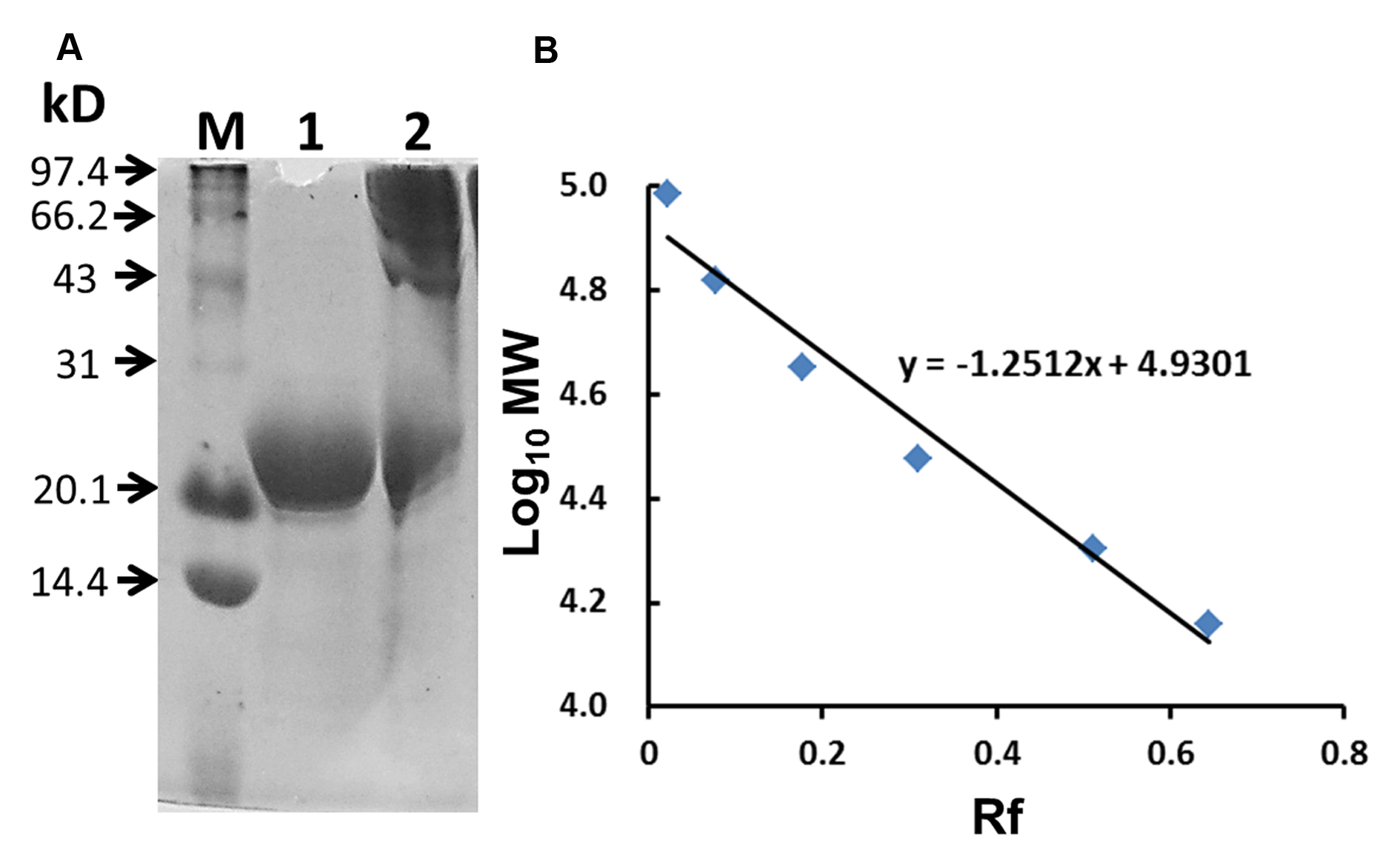

Supplement: Figure S2 — Estimation of molecular weight of the recombinant SBgLR monomer and oligomer. A, Native-PAGE analysis of the recombinant SBgLR. The native standard proteins were indicated by arrows. B, Molecular weight estimation for the oligomers. The molecular weights of the oligomers were 25.23 kDa, 51.00 kDa, 60.25 kDa, 65.90 kDa, 72.01 kDa, 76.36 kDa and 79.35 kDa, respectively. MW, Molecular weight; Rf, Protein migration. The molecular weight of the oligomer was estimated by the equation: log10MW = –1.2512x+4.9301 (x, protein migration). (TIF) [file pone.0060543.s002.tif]

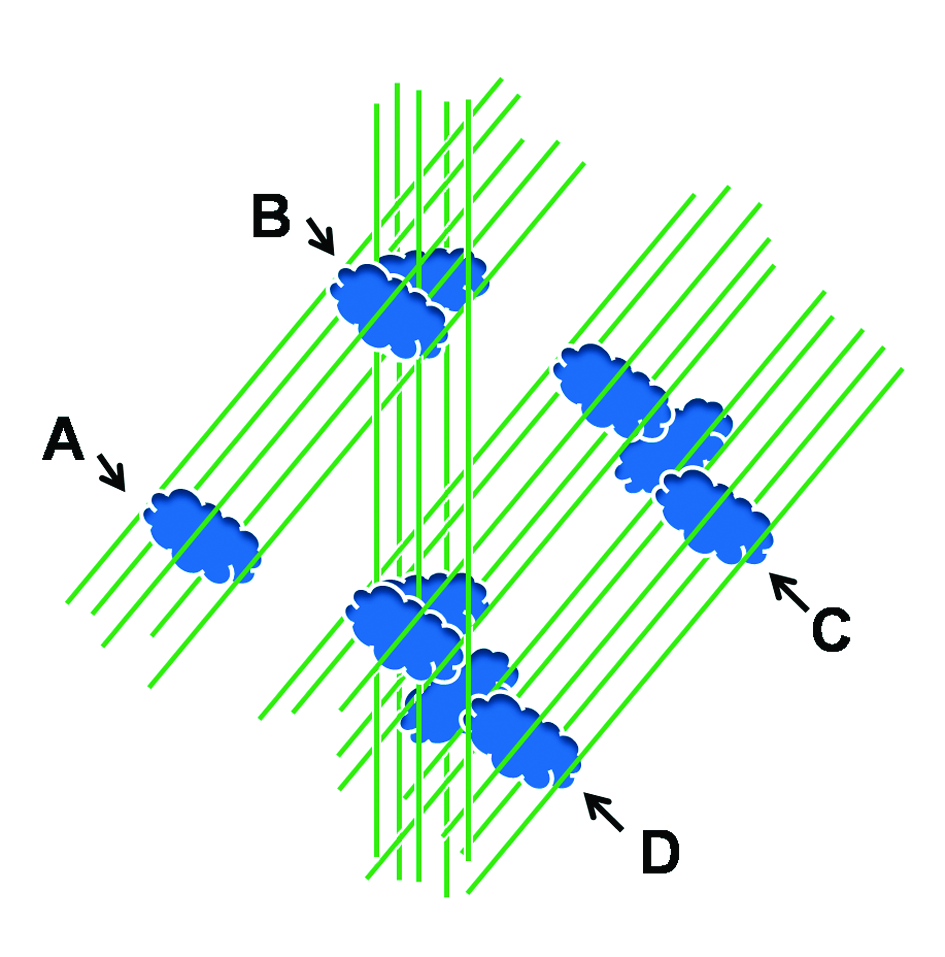

Supplement: Figure S3 — Schematic diagram of the probable roles of SBgLR monomers, dimers and oligomers in MT organization. A, The SBgLR monomers organized single MT into MT-bundles. B, The SBgLR dimers and oligomers make the MT-bundles cross-linked or reoriented. C and D, The SBgLR proteins stabilize the paralleled MT-bundles or make the MT-bundles meshing into network. (TIF) [file pone.0060543.s003.tif]
